# Response to Kellner, Espinoza, Gligorovic, and Sartorius

**DOI:** 10.1017/S0033291726103687

**Published:** 2026-03-24

**Authors:** Hamish Naismith, Jack Wilson, Harry Costello, Neil M Davies, Alexandra Pitman, Robert Howard

**Affiliations:** 1 Division of Psychiatry, University College London, London, UK; 2 North London NHS Foundation Trust, London, UK; 3 Department of Statistical Sciences, University College London, London, UK; 4 Department of Public Health and Nursing, Norwegian University of Science and Technology, Trondheim, Norway

Dear Editor,

We thank Kellner and colleagues for their thoughtful response (Kellner, Espinoza, Gligorovic, & Sartorius, Kellner, Espinoza, Gligorovic, & Sartorius, 2025) to our systematic review and meta-analysis (Naismith et al., 2025).

Kellner and colleagues note that we limited our review to studies with both an ECT arm and a comparator arm and that ‘this choice omits relevant studies in which an acute course of ECT is administered and suicidal thoughts and behaviors are measured before and after the intervention.’ We acknowledge that limiting our eligibility criteria to studies that had a comparator group will have resulted in some observational studies being excluded. However, we considered that inclusion of studies with a control group would be much less vulnerable to bias and would allow us to make more robust conclusions about the effectiveness of ECT.

The authors also state that ‘while Naismith et al. included 17 studies in their systematic review, their meta-analysis included only a subset of these.” They add that ‘it is not clear to us why all three outcomes [all-cause, suicide, and non-suicide mortality] would be needed to include a study for which one or two of the relevant outcomes were measured.’ In our paper, we noted that ‘not every study reported the number of events for all three outcomes (all-cause, suicide, and non-suicide mortality).” We should clarify that this sentence was a description of our results and not a criterion for excluding studies from our meta-analysis: reports indeed often only included one or two of our outcomes of interest and were not excluded from the meta-analysis on that basis. We apologize if this was unclear.

Kellner and colleagues cite the study by Rhee et al. (2021), which reported significant reductions in all-cause mortality until 1-year after ECT and suicide mortality until 90-days after ECT, as an example of a study that was ‘omitted from the meta-analyses, potentially altering the results.” However, we were unable to include this study in our meta-analysis: although they reported hazard ratios and numbers at risk at different timepoints, they did not report the actual numbers of events of our outcomes of interest.

The authors describe how ‘data from other studies compellingly demonstrate a benefit from ECT on all-cause and suicide mortality.’ In our meta-analyses, we found that ECT was associated with a significant reduction in all-cause mortality; for suicide mortality, we found there were ‘no differences in a consistent direction across all studies.” The authors question our conclusion that ‘it is possible that ECT has no effect on suicide mortality,” which they describe as an ‘unnecessarily negative assessment.” We added this statement in response to a reviewer comment and, while it is a theoretically possible explanation for our finding, we consider a much more probable explanation to be the high levels of heterogeneity between included studies. This interpretation would be consistent with the findings of two meta-analyses which, by only including patients with diagnoses of depression, had less clinically heterogenous populations compared to those included in our review. Odermatt et al. (2025), cited in our review, reported that ECT was associated with a statistically significant reduction in the odds ratio for suicide (OR 0.66, 95% CI 0.50–0.88) compared to treatment as usual. Kellner and colleagues also highlight the Chan et al. (2025) review and meta-analysis, which reported that ECT use was associated with reduced suicide mortality (RR = 0.67, 95% CI: 0.53–0.85) compared to no ECT use.

In further support of the view that high heterogeneity explains the results of our meta-analysis of suicide mortality, we note in our review that ‘studies that restricted the study population to patients with a higher severity of depression and accounted for confounding by indication through a wide range of covariates were more likely to show a reduction in suicide mortality.’

Taken together, we agree with Kellner and colleagues that these findings suggest that ECT is a safe and effective treatment when prescribed for appropriate indications.
